# P-1306. Priority Bacterial Pathogens Co-Resistance Networks Reveal Predictable Novel Pathways for Pan-Drug Resistance

**DOI:** 10.1093/ofid/ofaf695.1494

**Published:** 2026-01-11

**Authors:** Ramadhani Chambuso, Yehia Mohamed

**Affiliations:** Ajman University, Ajman, Ajman, United Arab Emirates; Ajman University, Ajman, Ajman, United Arab Emirates

## Abstract

**Background:**

The rising global concern for the development of pan-drug resistant (PDR) infections is alarming. We mapped the existing structural high antimicrobial co-resistance networks for potential novel pathways for resistance trajectories that may lead to PDR across WHO priority bacterial pathogens.

**Figure 1.**
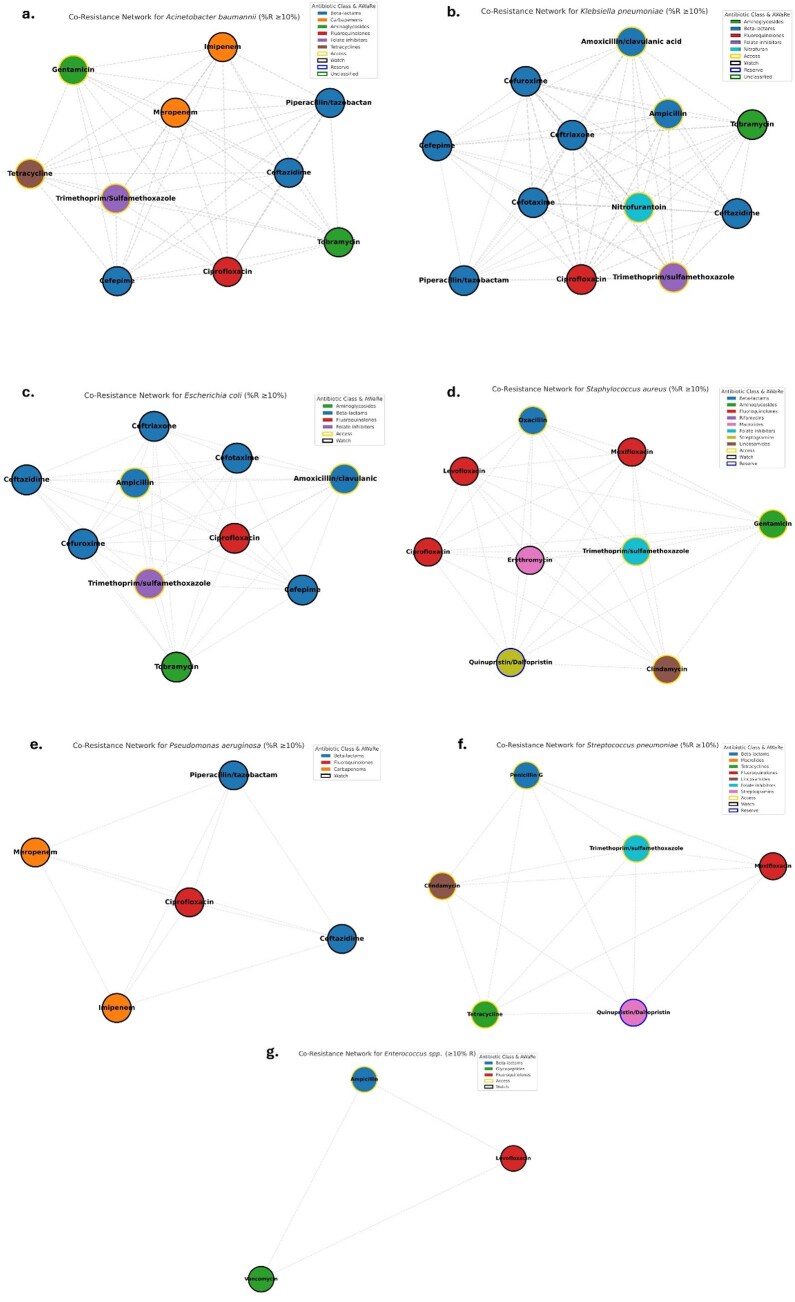
Co-resistance network analysis (% Resistance > 10) for the WHO bacterial priority pathogens.

**Figure 2.**
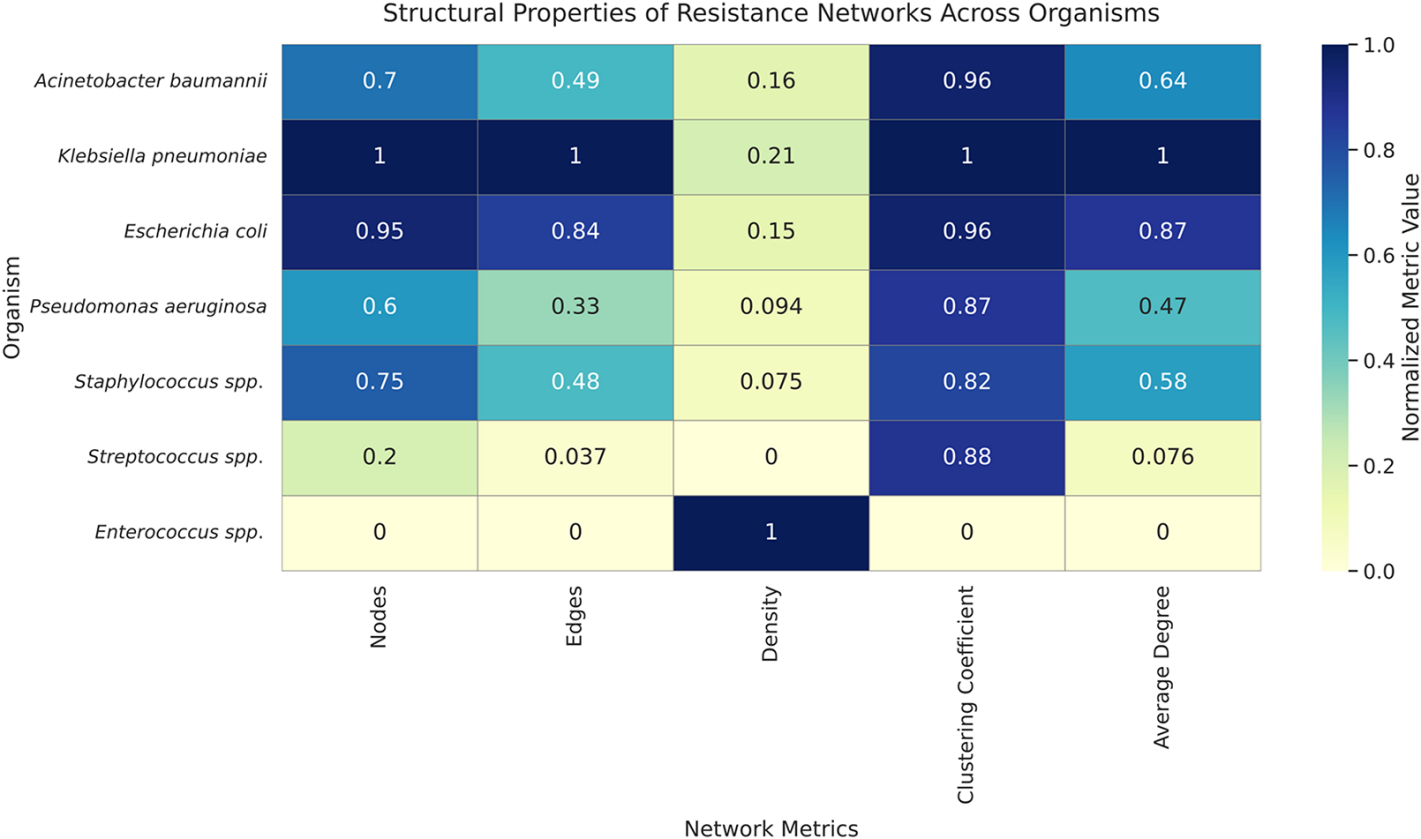
Heat map showing co-resistance network matrices for each WHO priority bacterial pathogen in the network analysis

**Methods:**

We analysed 151,358 clinical isolates data collected over 14 years (2010–2023) from different hospitals in the UAE with their AMR profiles. Pathogen-drug co-resistance networks were constructed using antibiotic pairs with ≥10% resistance. Nodes were annotated by drug class and WHO AWaRe classification. Network structures were quantified by nodes, edges, density, clustering and degree.

**Figure 3.**
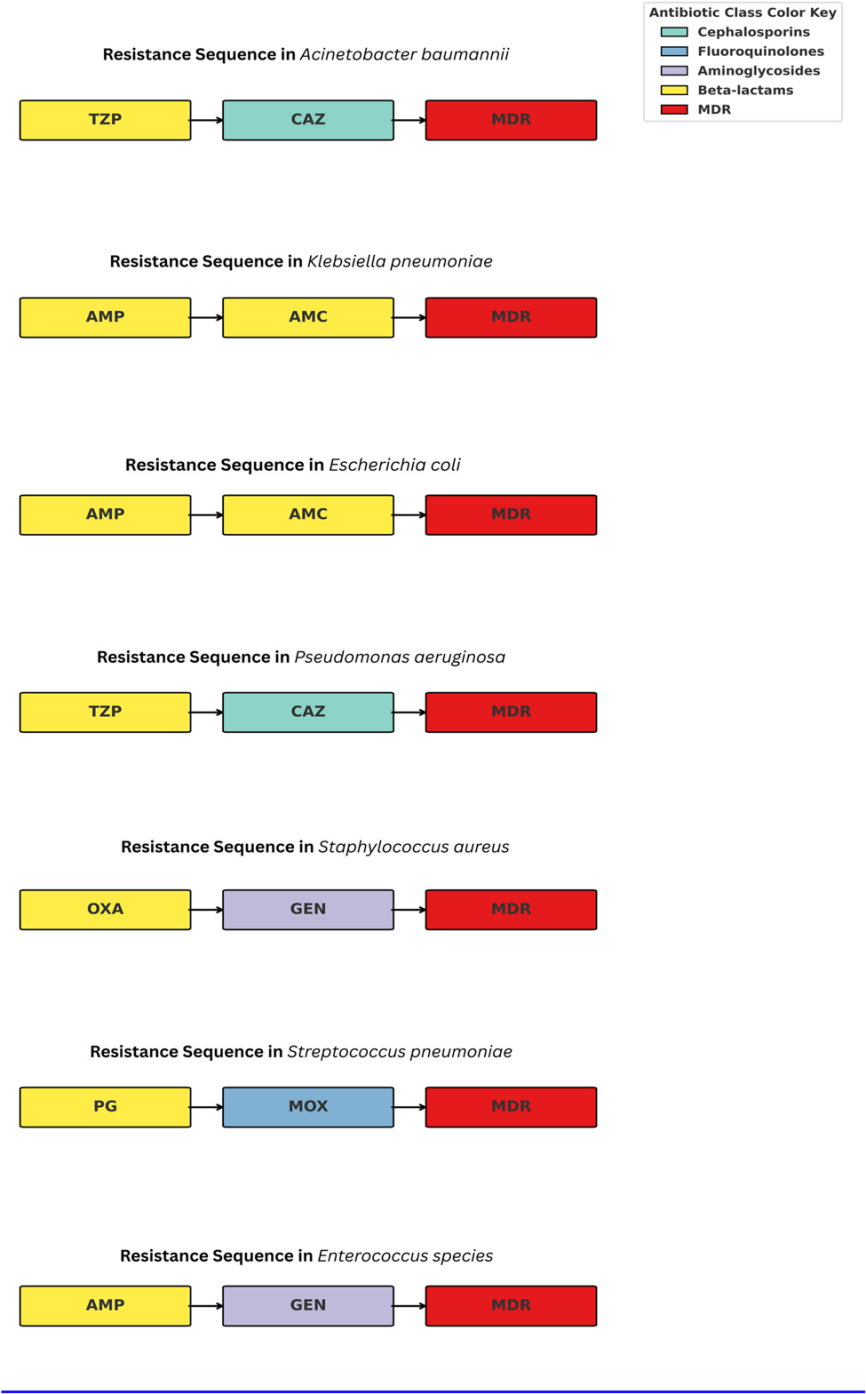
Resistance pathways derived from the co-resistance network matrices for drug pairs with min (%Resistance 1, %Resistance 2) ≥ 10%, retained as weighted edges showing pathways to MDR for each pathogen.

**Figure 4.**
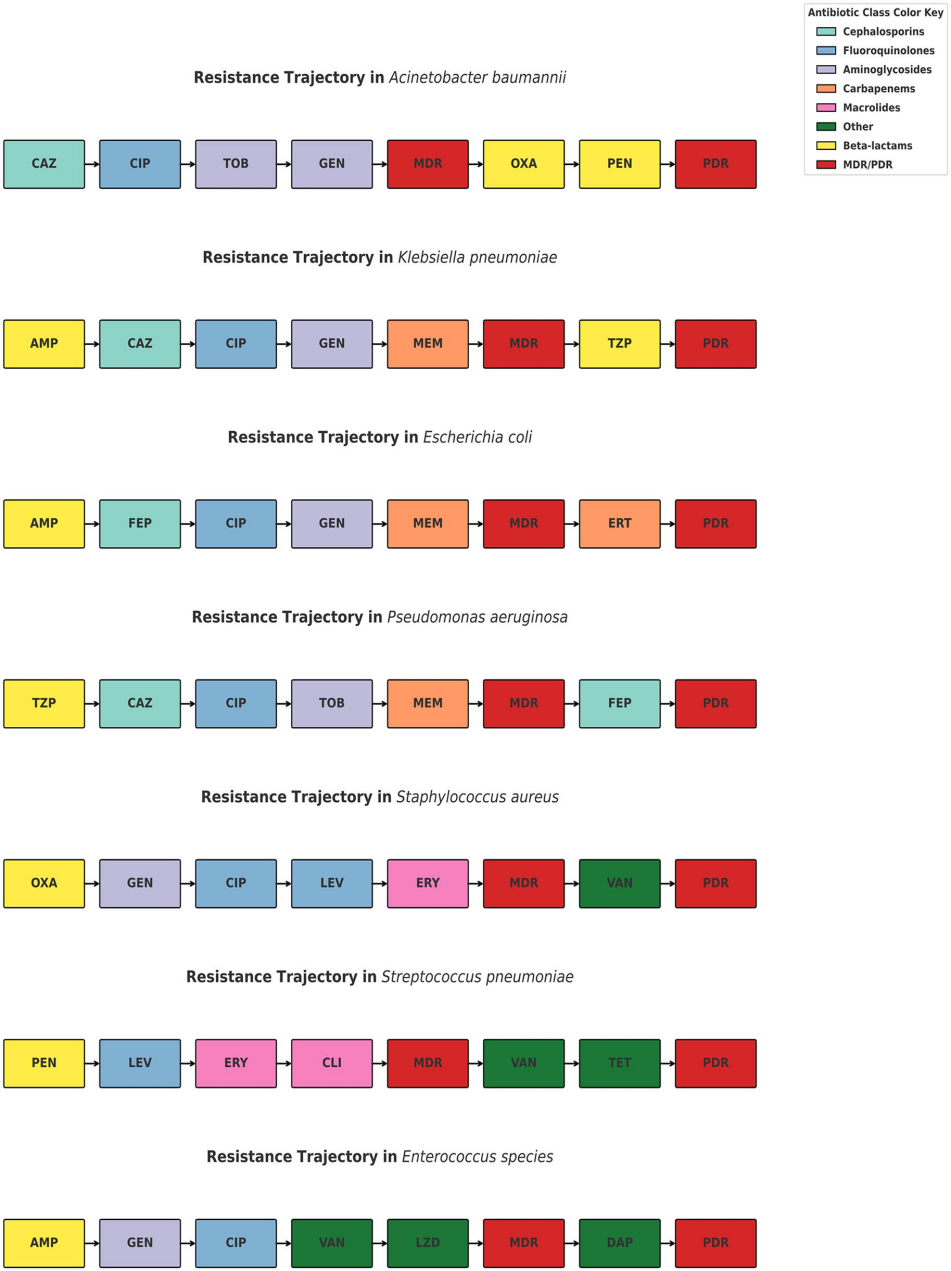
Universal novel resistance pathways highlighting key resistance trajectories towards MDR and PDR.

**Results:**

The resistance trajectories revealed species-specific antibiotic resistance pathways ending to MDR/PDR. The co-resistance network-based analysis captured statistically inferred transitions between drugs co-occurring ≥10%. *K. pneumoniae*, *E. coli*, and *A. baumannii* exhibited highly interconnected networks with extensive beta-lactam and aminoglycoside co-resistance. PDR trajectory pathways for each organism typically passed through MDR transition nodes before converging on carbapenem, vancomycin, or linezolid resistance. Transition modelling confirmed early fluoroquinolone resistance as a shared gateway to MDR, followed by organism-specific paths culminating in PDR. There were consistent resistance progression patterns that offered universal complementary frameworks for anticipating resistance evolution and optimizing empiric therapy.

**Conclusion:**

Drug resistance evolution is predictable and convergent, not random. Despite species differences, resistance followed a convergent topology anchored on early fluoroquinolone resistance and structured progression to PDR. This framework enables early identification of high-risk resistance pathways and supports precision antimicrobial stewardship

**Disclosures:**

All Authors: No reported disclosures

